# The Contribution of Eight Modifiable Risk Factors to Dementia Prevalence in Jamaica

**DOI:** 10.1093/geroni/igaa057.1210

**Published:** 2020-12-16

**Authors:** Kimberly Ashby-Mitchell, Denise Eldemire-Shearer

**Affiliations:** University of the West Indies, Kingston, Jamaica

## Abstract

The number and proportion of dementia cases in Jamaica attributable to eight modifiable lifestyle factors was calculated using factor analysis in Stata and Levin’s Attributable Risk formula (hypertension, diabetes mellitus, depression, low educational attainment, smoking, overweight, obesity and vision problems). Four sources of data were used: (1) risk factor prevalence data were obtained from the Jamaica Health and Lifestyle Survey, 2008, (2) relative risk data were sourced from published meta-analyses, (3) factor analysis in Stata version 15 was performed to estimate shared variance among risk factors utilising data from the Health and Social Status of Older Persons in Jamaica Study and, (4) estimated prevalence of dementia in Jamaica in 2012 was obtained from a 2018 publication by Shearer et al. Obesity was found to be responsible for the largest proportion of dementia cases (13.94%; 2508 cases) followed by hypertension (10.64%; 1917 cases), low educational attainment (7.21%; 1299 cases), overweight (6.42%; 1156 cases), smoking (3.90%; 702 cases), diabetes (3.38%; 608 cases), depression (1.00%; 181 cases) and impaired vision (0.002%; 34 cases%). Using this largely theoretical model, the eight factors examined account for approximately 46.0% of dementia cases. Dementia risk reduction programs that target even one of these factors has the potential to result in significant reduction in future dementia prevalence as they are all interrelated. In future work, as these data become available, the contribution of mid-life obesity, mid-life hypertension and physical inactivity will also be taken into consideration.

